# Nesfatin-1 in the dorsal raphe nucleus influences visceral sensitivity via 5-HT neurons in male maternally separated rats

**DOI:** 10.1038/s41598-018-27592-x

**Published:** 2018-06-19

**Authors:** Hui-Ai Zhang, Nan Sang, Xian Ge, Qian Huang, Xue-Liang Li, Jie Sha

**Affiliations:** 1 Department of Gastroenterology, the First Affiliated Hospital of Nanjing Medical University, Nanjing, Jiangsu Province 210029 China; 2 Department of Gastroenterology, Jingjiang People’s Hospital, Jingjiang, Jiangsu Province 214500 China

## Abstract

Nesfatin-1, a satiety molecule processed from nucleobindin2 (NUCB2), is implicated in visceral hypersensitivity in rats and colocalized with 5-hydroxytryptamine (5-HT) in the dorsal raphe nucleus (DRN). Maternal separation (MS) in rats contributes to visceral hypersensitivity via elevated expression of 5-HT in the DRN. Intracerebroventricular injection of nesfatin-1 activates DRN 5-HT neurons. In this study, A model of visceral hypersensitivity was developed by subjecting rats to MS. Colorectal distension was used to detect visceral sensitivity, which was evaluated by abdominal withdrawal reflex (AWR) scores and electromyogram (EMG) magnitude. MS rats exhibited higher AWR scores and EMG magnitude compared with controls. The numbers of nesfatin-1- and tryptophan hydroxylase (TPH, the rate-limiting enzyme for 5-HT synthesis)-positive cells in the DRN were significantly elevated accordingly. Visceral hypersensitivity was significantly alleviated in MS rats treated with intra-DRN administration of anti-nesfatin-1/NUCB2, accompanied by decreased expression of 5-HT and TPH in the DRN, compared with the vehicle-treated group. In contrast, intra-DRN administration of nesfatin-1 into normal adult rats induced visceral hypersensitivity, which correlated with elevated expression of 5-HT and TPH in the DRN. In conclusion, Nesfatin-1 has critical effects on visceral hypersensitivity; the underlying mechanisms might be related to the activation of DRN 5-HT neurons.

## Introduction

Irritable bowel syndrome (IBS) is a chronic gastroenterological disease of uncertain etiology marked by abdominal pain or discomfort and disorder of bowel movements. Albeit a common disorder, IBS exerts a negative impact on the quality of life and work productivity of patients^1^. While the pathophysiological mechanism of IBS remains incompletely elucidated, visceral hypersensitivity has been established as a key feature of IBS^2,3^.

Several neurotransmitters are linked to the pathophysiology of visceral hypersensitivity. The neurotransmitter 5-HT is synthesized in serotonergic neurons of the central nervous system(CNS) and possesses an important role in visceral hypersensitivity in patients with IBS^4^. Altered 5-HT signaling of the central nervous system contributes to the development of IBS—as indicated by the therapeutic effectiveness of both 5-HT3 receptor antagonists and tricyclic antidepressants^5,6^. Enteric 5-HT interacts with 5-HT_3_ receptors via afferent fibers that connect the gut with the central stress circuit (paraventricular nucleus, hippocampus, amygdala, locus coeruleus, and the raphe nucleus)^4^. These cerebral areas are abnormally activated due to chronic exteroceptive stress, leading to a local release of 5-HT, which may facilitate visceral pain processing via efferent fibers, and further result in visceral hypersensitivity^7^.

The 5-HT system of the brain is mainly concentrated in the raphe nucleus—the dorsal raphe nucleus (DRN) is the site of 5-HT synthesis in the brain^8^. Although the DRN is not the only participant of pain processing, it has a critical role in visceral pain modulation^9^. Ren *et al*. reported that visceral hypersensitivity in rats subjected to maternal separation (MS) is associated with elevated 5-HT and c-fos expression in the DRN in response to stress^10^. In contrast, downregulation of DRN serotonergic activity through electroacupuncture attenuates visceral hypersensitivity in MS rats^11^.

Nesfatin-1 is a anorectic neuropeptide processed from nucleobindin2 (NUCB2). Besides its homeostatic functions associated with food intake, mounting evidence indicates its role in the mediation of the stress response^12^. Central nesfatin-1 neurons are stimulated after restraint stress. Intracerebroventricular administration of nesfatin-1 activates corticotropin-releasing hormone (CRH), noradrenaline and 5-HT neurons, and induces the HPA axis^13^.

Previous studies by these authors demonstrated that nesfatin-1 is implicated in visceral hypersensitivity^14^. Furthermore, upregulated expression of nesfatin-1/NUCB2 in the amygdala results in visceral hypersensitivity in an MS model in rats^15^. Nesfatin-1 is colocalized with 5-HT in the DRN^16^. Rats exposed to MS manifested persistent IBS-like visceral hypersensitivity via elevated 5-HT expression in the DRN^10^. Furthermore, an intracerebroventricular injection of nesfatin-1 activates DRN 5-HT neurons^13^. These previous studies indicate that nesfatin-1 in the DRN may also have effects on visceral hypersensitivity, potentially through activation of DNR 5-HT neurons. In this study, we sought to determine the effect of nesfatin-1 in the DRN on visceral sensitivity using a maternally separated rat model of IBS. Antibodies against 5-HT and tryptophan hydroxylase (TPH, the rate-limiting enzyme for 5-HT synthesis) were used as markers of 5-HT neurons. Effects of nesfatin-1 on visceral hypersensitivity and DRN 5-HT neurons were studied.

## Material and Methods

### Animals and housing

Pregnant Sprague–Dawley rats were obtained from the Animal Core Facility, Nanjing Medical University (Nanjing, China), and were caged separately under controlled temperature (20 °C), with a 12 h/12 h light-dark cycle, and with free access to food and water. All experiments were carried out in compliance with the guidelines set forth by the Institutional Council on Animal Care and with approval from the Ethics Committee of Nanjing Medical University.

### Maternal separation

Female newborn pups were culled at the birth time. Male newborn rats were randomly allocated either to the MS group or the normally handled (NH) group. Each group contained three dams and 6–8 pups per litter. The MS protocol was applied as reported previously^17^. Briefly, pups in the MS group were placed into individual plastic cages with sawdust bedding maintained at 22 °C in a separate room to isolate them from their mothers for 3 h daily (8:00–11:00) from postnatal days 2 to 21, whereas pups in the NH group were undisturbed. After weaning on Day 22, 22 male pups in the MS group and 20 male pups in the NH group were housed by treatment group with 5 rats per cage until surgery at 8 weeks of age.

### Experimental design

At the beginning of the experiment, when rats in the MS and NH group were 8 weeks of age, six rats in each group were randomly selected to evaluate visceral sensitivity. Then the remaining rats were prepared for DRN microinjection, the MS group was divided into 2 groups as anti-nesfatin-1/NUCB2 and vehicle, while NH group was designated as nesfatin-1 and vehicle respectively.

### Evaluation of visceral sensitivity

Visceral responses to different grades of colorectal distension (CRD) pressures were quantified by AWR scores and electromyographic activities. Briefly, rats were sedated with isoflurane in a sealed cage. A distension balloon—made from a latex glove finger (5 cm in length) attached to a suction tube via a Y connector to a syringe pump and a sphygmomanometer—was inserted intrarectally 8 cm via the anus and secured to the tail. Rats were maintained in small Lucite cubicles(20 cm × 6 cm × 8 cm) and allowed to wake up and adapt for 20 min. CRD was carried out at graded intensities of 20, 40, 60, and 80 mmHg as previously described^18^. Each CRD stimulation period was sustained for 20 s, followed subsequently by a 2-min rest. AWR responses were measured by blinded observers who assigned AWR scores as follows: (1) normal behavior without response; (2) contraction of abdominal muscles; (3) lifting of abdominal wall; and (4) body arching and lifting of pelvic structures. To obtain EMG measurements of visceromotor responses, after the animals were anesthetized with 10% chlorohydrate (0.3 ml/100 g body weight, intraperitoneally), a pair of electrodes was stitched into the obliquus externus abdominis, tunneled subcutaneously, then exteriorized, and finally secured at the back of the neck. Rats were individually housed and recovered for 1 week after surgery. EMG signals were amplified and digitized with a PowerLab system (AD Instruments, New South Wales, Australia) and analyzed using Lab Chart 7. EMG activity was identified by the increase of the area under the curve (AUC) during 20 s of CRD over the baseline AUC (20 s before distension).

### Stereotaxic cannulation

**Figure 1:**
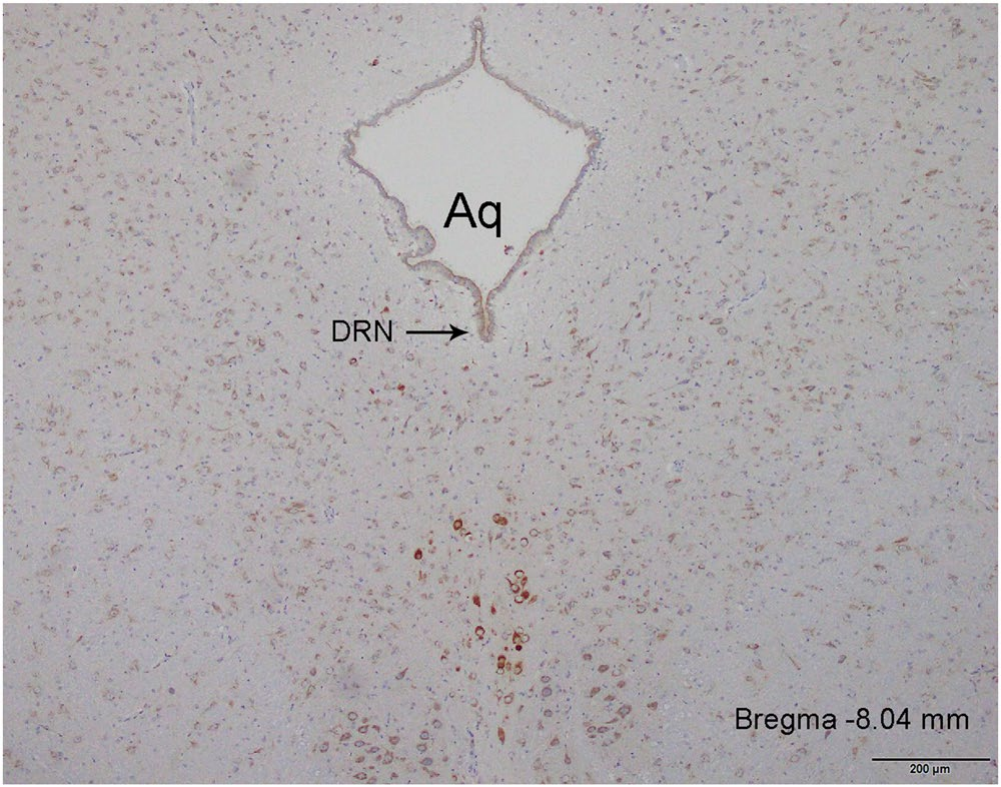
Photomicrographs of representative cannula placements in the DRN. Sections were according to Paxinos and Watson (2007). Aq, aqueduct; DRN, dorsal raphe nucleus.

Under anesthesia with 10% chlorohydrate (0.3 ml/100 g body weight, intraperitoneally), we implanted a 26-gauge guide cannula into the DRN at the following coordinates, AP = −8.0; L = 0.0, and DV = −5.8 (Paxinos and Watson)^19^_,_ and then it was held in place with stainless steel screws and dental cement. For microinjection, an injection cannula (29 gauge), which was designed to protrude 1 mm beyond the guide, was inserted. Cannula/injection-site verification was done at the end of the experiment. Only rats having cannulas within the scope of the DRN were included in the data analysis. 1 animal in the MS group and 2 animals in the NH group with incorrect DRN targeting were excluded from the final analysis.The cannula/injection location is shown in Fig. 1. Finally, two electrodes were implanted in the external oblique muscle as described above. Rats were individually housed, treated postoperatively for three days with analgesic dipyrone (100 mg/Kg) intramuscularly and recovered for 1 week after surgery.

### Intra-DRN microinjection and visceral sensitivity testing

Seven days after surgery, intra-DRN injections were administered in accordance with the Paxinos and Watson atlas^19^. Rats in the MS model were microinjected with anti-nesfatin-1/NUCB2 (1 µL, 10 µg; Santa Cruz Biotechnology, Santa Cruz, CA, USA) or vehicle (1 µL, sterile water) into the DRN; then, abdominal withdrawal reflex (AWR) scores and magnitude of electromyogram (EMG) were recorded 2 h later. While normal rats were microinjected with nesfatin-1 (1 µL, 50 pmol; Phoenix Pharmaceuticals, Burlingame, CA, USA) or vehicle (1 µL, sterile water) into the DRN, nesfatin-1 was injected daily for 7 consecutive days^15^ then visceral sensitivity was detected at the 7th day.

### Tissue preparation and immunohistochemistry

Immediately after completion of the experiments, under deep anesthesia with 10% chlorohydrate (0.3 ml/100 g body weight), rats were perfused with saline followed by 4% paraformaldehyde. Brains were quickly removed, immersed in the same fixative, and stored at 4 °C for 3–5 days. Thereafter, brains were paraffin embedded, and sections were cut at a thickness of 3 μm. Immunohistochemistry was conducted following antigen retrieval using the Envision method. All sections were submerged in citrate buffer (pH 6.0) and heated in a pressure cooker for antigen retrieval. Slides were then incubated with a rabbit polyclonal anti-nesfatin-1 antibody (1:2400; cat#. H-003–22, Phoenix Pharmaceuticals), a mouse monoclonal anti-TPH antibody (1:1000; cat#. T0678, Sigma-Aldrich, USA), or a rabbit anti-5-HT antibody (1:8000; cat#. S5545, Sigma-Aldrich) at room temperature (RT) for 2 h. Secondary Envision Flex/HRP (horseradish peroxidase; Dako, 20 min) was added for signal amplification. Next, immunoreactivity was visualized with 3,3′-diaminobenzidine (DAB, Dako) before brief counterstaining with hematoxylin. The slides were dehydrated and mounted. Negative controls were set by omitting the primary antibody.

### Quantitative morphometry

Nesfatin-1-, 5-HT-, or TPH-immunopositive cells in the DRN were counted manually at 10 × 10 magnification. Clear demarcation existed between all counted cells and background staining. Cell counting was carried out by two blinded observers. Immunopositive cells in the DRN were counted in 3 non-adjacent coronal sections across DRN (bregma −7.32 mm ~ −8.28 mm) and averaged per animal(6 animals per treatment). The mean number of nesfatin-1-, 5-HT-, or TPH-positive cells was calculated for each group, and a standard error of the mean was determined.

### Data Analysis and Statistics

All data are presented as the mean ± SE, Two-way repeated-measures ANOVA with Bonferroni post-hoc testing are used for colonic sensitivity analysis. Other data were analyzed by the Student’s t-test. Significance was defined as p < 0.05.

### Data availability

The images and datasets generated and analyzed during the current study are available from the corresponding author on reasonable request.

## Results

### Rats exposed to MS exhibited persistent visceral hypersensitivity

**Figure 2:**
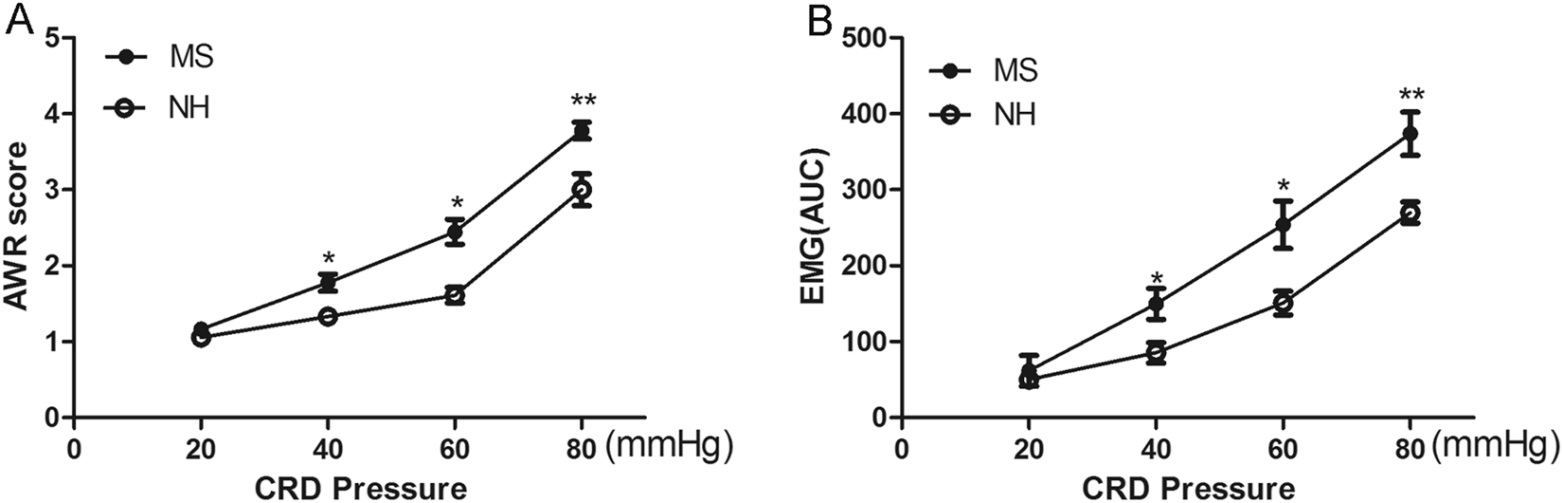
Evaluation of visceral sensitivity in MS and NH rats when adults. (**A)** Abdominal withdrawal reflex (AWR) scores under graded colorectal distension pressures. **(B)** Area under the curve(AUC) of Electromyographic(EMG) activity in the external oblique muscle in response to graded distension pressures. Rats exposed to MS exhibited higher mean AWR scores **(**Fig. 2A**)** and enhanced EMG magnitude at strengths of 40, 60, and 80 mmHg CRD pressure when compared to the NH group [^*^p < 0.05, ^**^p < 0.01] (*n* = 6 for each group).

To assess visceral sensitivity, we observed AWR scores and EMG magnitude in response to graded strengths of CRD pressures in MS and control rats at 8 weeks of age. MS rats exhibited higher AWR score [*p* = 0.01; *p* = 0.01; *p* = 0.009; *n* = 6 for each group] (Fig. 2A**)** and more intensive EMG magnitude [*p* = 0.035; *p* = 0.015; *p* = 0.009*; n* = 6 for each group] (Fig. 2B) at strengths of 40, 60, and 80 mmHg CRD pressure when compared to NH rats.

### Nesfatin-1/NUCB2 and TPH immunoreactivity in the DRN of MS rats

**Figure 3:**
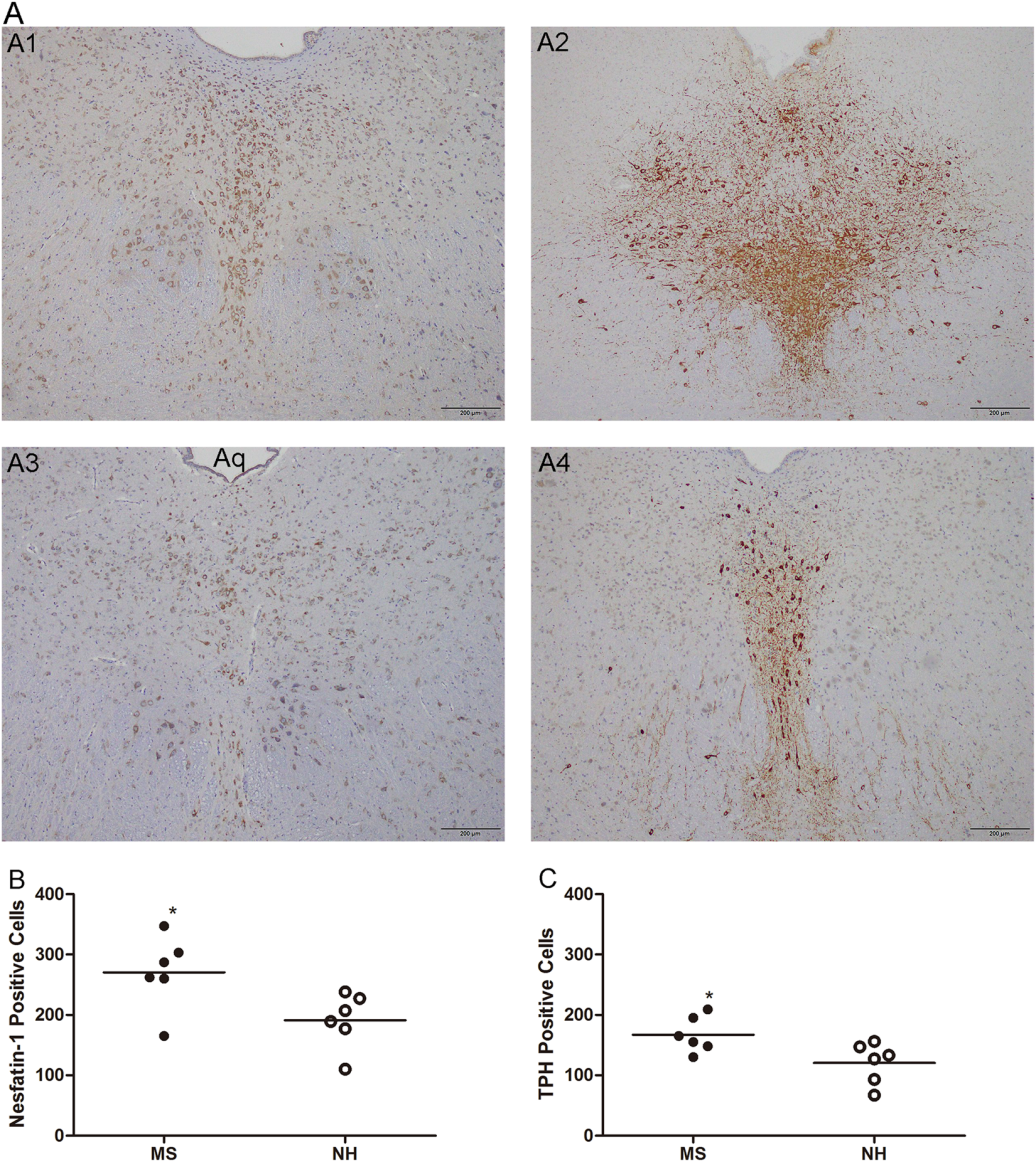
Nesfatin-1 and TPH immunoreactivity in the DRN of MS and NH rats. (**A**) Photomicrographs (100 × magnification) of nesfatin-1 and TPH-immunopositive cells in the DRN of MS and NH rats; A1: MS–nesfatin-1; A2: MS–TPH; A3: NH–nesfatin-1; and A4: NH–TPH. (**B**) Number of nesfatin-1-immunopositive cells in the DRN of MS and NH rats. (**C**) Number of TPH-immunopositive cells in the DRN of MS and NH rats. MS rats showed significantly elevated numbers of nesfatin-1- and TPH-immunopositive cells in the DRN [^*^p < 0.05] (*n* = 6 for each group).

The MS rats showed an obvious and significant enhancement of nesfatin-1 and TPH immunoreactivity, compared to NH rats, in all sections of the DRN (representative images in Fig. 3A**)**. Image analysis quantification showed that the expression of nesfatin-1 (270.67 ± 60.82, in the MS group, vs 191.33 ± 45.87, in the NH group, *p* = 0.029; *n* = 6 for each group) or TPH (167.00 ± 29.75, in the MS group, vs 120.50 ± 33.99, in the NH group, *p* = 0.03; *n* = 6 for each group) was significantly elevated in the DRN of MS rats, as shown in Fig. 3B,C.

### Intra-DRN administration of anti-nesfatin-1/NUCB2 reduced visceral hypersensitivity

**Figure 4:**
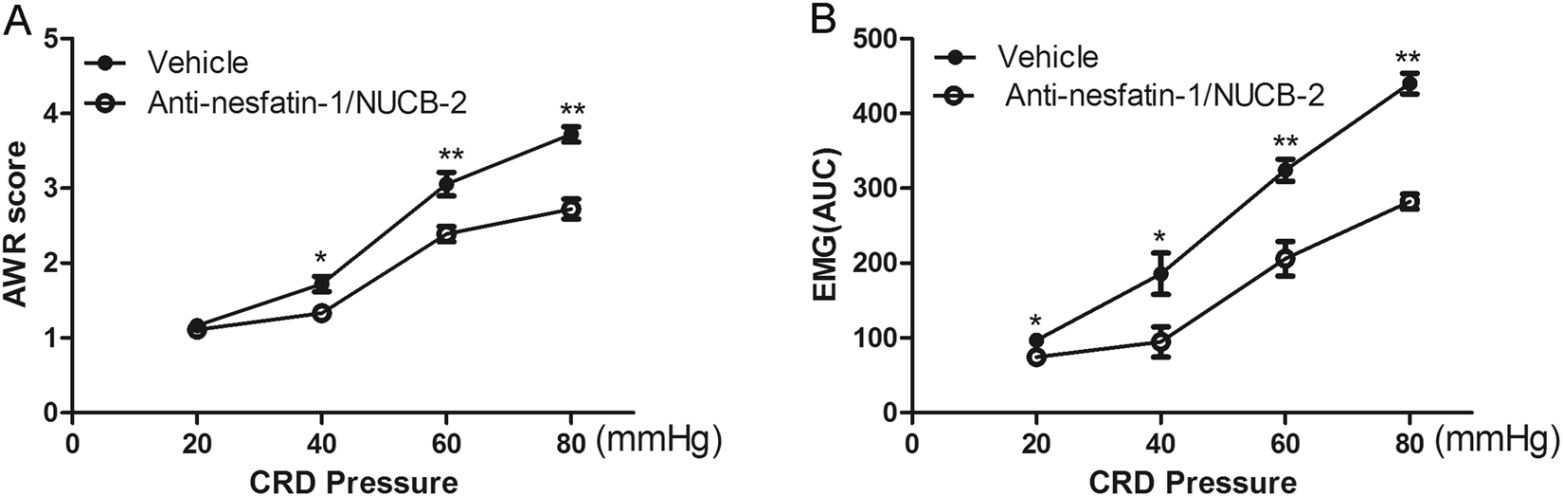
Effect of anti-nesfatin-1/NUCB-2 on visceromotor response to CRD in MS rats. (**A**) AWR scores under graded colorectal distension pressures. (**B**) AUC of EMG activity in response to graded distension pressures. Intra-DRN injection of anti-nesfatin-1/NUCB2 in MS rats resulted in a significant decline in the mean AWR scores at strengths of 40, 60, and 80 mmHg distension pressures (Fig. 4A**)** and EMG magnitude at all distension pressures, compared with the vehicle group [^*^p < 0.05, ^**^p < 0.01] (*n* = 6 for each group).

To evaluate whether DRN nesfatin-1 is related to the regulation of visceral sensitivity, we observed AWR scores and EMG magnitude in response to graded strengths of CRD pressures in MS rats treated with anti-nesfatin-1/NUCB2 or vehicle. We found that intra-DRN injection of anti-nesfatin-1/NUCB2 in MS rats resulted in a significant decline in the AWR scores at 40, 60, and 80 mmHg distension pressures [*p* = 0.015; *p* = 0.005; *p* < 0.001; *n* = 6 for each group] (Fig. 4A**)** and EMG magnitude at all distension pressures [*p* = 0.044; *p* = 0.024; *p* = 0.002; *p* < 0.001; *n* = 6 for each group] (Fig. 4B), as compared with vehicle-treated rats.

### Effects of intra-DRN administration of anti-nesfatin-1/NUCB2 on 5-HT neurons

**Figure 5:**
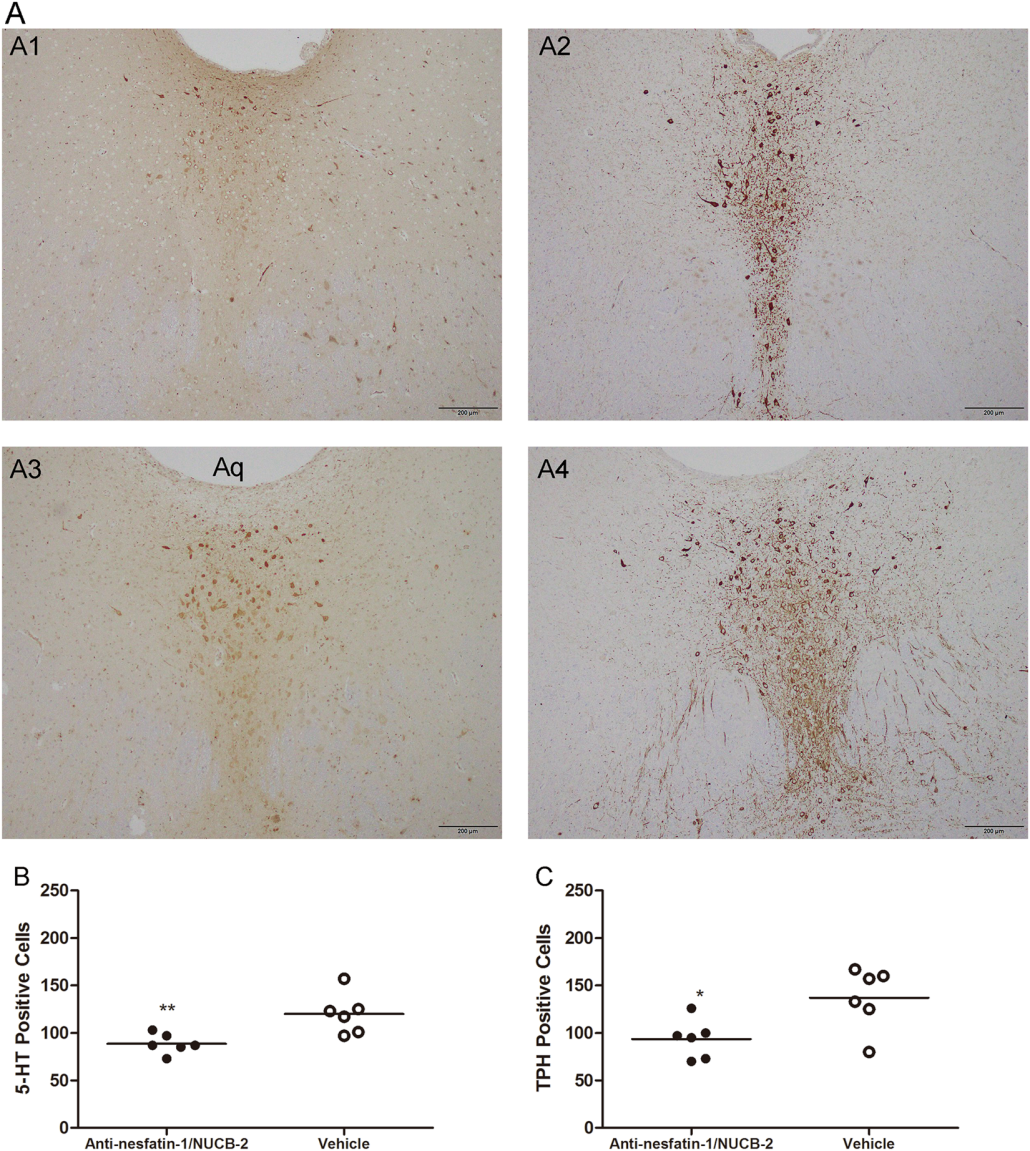
5-HT and TPH immunoreactivity in the DRN of MS rats treated with anti-nesfatin-1/NUCB-2 or vehicle. (**A**) Representative examples of sections processed for 5-HT or TPH immunostaining from MS rats microinjected with anti-nesfatin-1/NUCB-2 or vehicle into the DRN; A1: MS + anti-nesfatin-1/NUCB-2–5-HT; A2: MS + anti-nesfatin-1/NUCB-2–TPH; A3: MS + vehicle–5-HT; and A4: MS + vehicle–TPH. (**B**)Number of 5-HT-immunopositive cells in the DRN of anti-nesfatin-1/NUCB-2-or vehicle-microinjected MS rats. (**C**)Number of TPH-immunopositive cells in the DRN of anti-nesfatin-1/NUCB-2-or vehicle-microinjected MS rats. Anti-nesfatin-1/NUCB2 microinjection resulted in a significant reduction in the number of 5-HT-and TPH-immunopositive cells in the DRN of MS rats. [^*^p < 0.05, ^**^p < 0.01] (*n* = 6 for each group).

We observed 5-HT and TPH immunoreactivity in the DRN after application of anti-nesfatin-1/NUCB2 in MS rats. Representative images are presented in Fig. 5A. Intra-DRN injection of anti-nesfatin-1/NUCB2 led to a significant reduction of 5-HT- (88.67 ± 10.38, in the anti-nesfatin-1/NUCB2 antibody group, vs 120.00 ± 21.46, in the vehicle group, *p* = 0.009; *n* = 6 for each group) or TPH-(93.50 ± 20.42, in the anti-nesfatin-1/NUCB2 group, vs 137.00 ± 32.37, in the vehicle group, *p* = 0.019; *n* = 6 for each group) immunopositive cells in the DRN of MS rats (Fig. 5B,C).

### Effects of intra-DRN injection of nesfatin-1 on visceral sensitivity in normal rats

**Figure 6:**
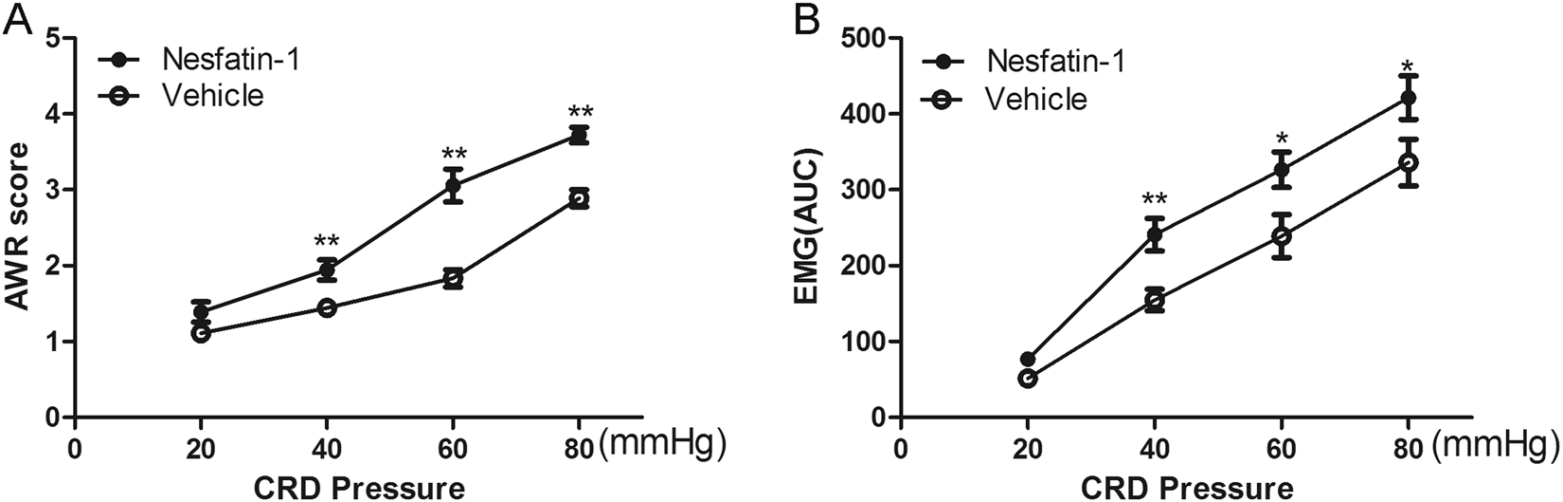
Effect of nesfatin-1 on visceromotor response to CRD in NH rats. (**A**) AWR scores under graded distension pressures. (**B**) AUC of EMG activity in response to graded distension pressures. Intra-DRN injection of nesfatin-1 in NH rats resulted in significantly increased AWR scores and EMG magnitude at 40, 60, and 80 mmHg distension pressures with the vehicle group. [^*^p < 0.05, ^**^p < 0.01] (*n* = 6 for each group).

To identify whether DRN nesfatin-1 contributes to the development of visceral hypersensitivity, we observed AWR scores and EMG magnitude in response to graded strengths of CRD pressures in normal rats treated with nesfatin-1 (50 pmol) or vehicle. We found that intra-DRN injection of nesfatin-1 in normal rats resulted in significantly elevated AWR scores [*p* = 0.008; *p* = 0.001; *p* < 0.001; *n* = 6 for each group] (Fig. 6A) and EMG magnitude [*p* = 0.007; *p* = 0.039; *p* = 0.032; *n* = 6 for each group] (Fig. 6B) at 40, 60, and 80 mmHg distension pressures, relative to vehicle-treated rats.

### Effects of intra-DRN injection of nesfatin-1 on 5-HT neurons

**Figure 7:**
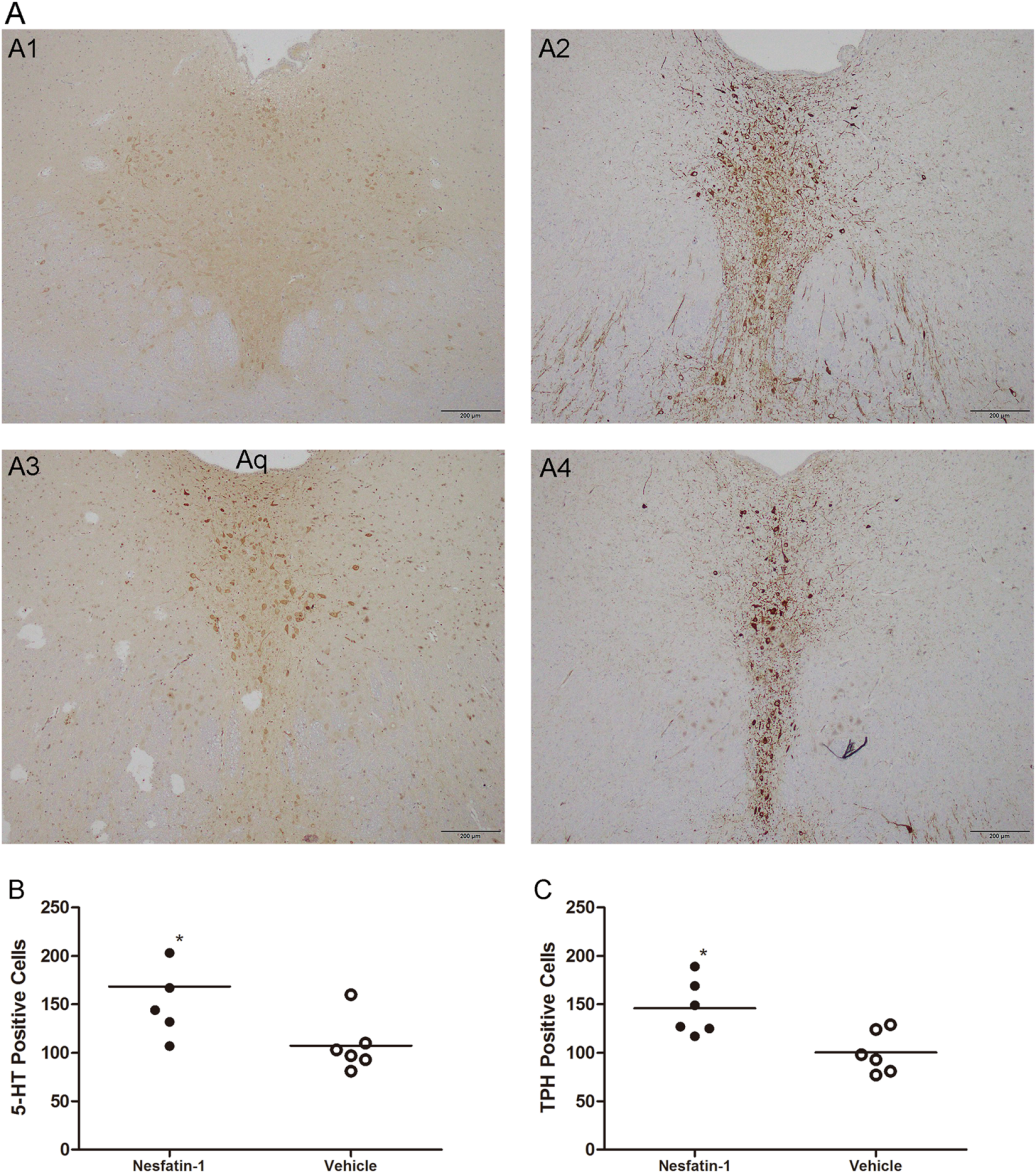
5-HT and TPH immunoreactivity in the DRN of NH rats treated with nesfatin-1 or vehicle. (**A**) Representative examples of sections processed for 5-HT or TPH immunostaining from NH rats microinjected with nesfatin-1 or vehicle into the DRN; A1: NH + nesfatin-1–5-HT; A2: NH + nesfatin-1–TPH; A3: NH + vehicle–5-HT; and A4: NH + vehicle–TPH. (**B**) Number of 5-HT-immuno-positive cells in the DRN after nesfatin-1 or vehicle microinjection. (**C**) Number of TPH-immunoreactive cells in the DRN after nesfatin-1 or vehicle microinjection. Nesfatin-1 microinjection caused a significant elevation in the number of 5-HT and TPH-immunoreactive cells in the DRN of NH rats [^*^p < 0.05] (*n* = 6 for each group).

We observed 5-HT and TPH immunoreactivity in the DRN after application of nesfatin-1 in normal rats. Representative images are presented in Fig. 7A. After the intra-DRN injection of nesfatin-1 in normal rats, the number of 5-HT- (168.50 ± 54.62, in the nesfatin-1 group, vs 107.33 ± 27.59, in the vehicle group, *p* = 0.034; *n* = 6 for each group) or TPH- (146.00 ± 28.39, in the nesfatin-1 group, vs 100.33 ± 21.72, in the vehicle group, *p* = 0.011; *n* = 6 for each group) immunopositive cells in the DRN was significantly increased as compared with vehicle-injected rats in all sections of the DRN (Fig. 7B,C).

## Discussion

IBS is recognized as a stress-related disorder^20,21^. Stress scores are higher in patients with IBS than with other functional bowel disorders, and stress is also significantly correlated with IBS symptoms^21^. A history of early adverse life events is more likely to result in IBS^22^. In our study, we used neonatal MS to establish a rat model of visceral hypersensitivity that mimics IBS. MS rats exhibited higher AWR scores and more intensive EMG magnitude at strengths of 40, 60, and 80 mmHg CRD pressure relative to controls. The present results indicate that MS produced persistent visceral hypersensitivity. Early-life stressors such as trauma, assault, disaster, various types of abuse, and chronic illness of a parent are related to IBS symptoms^23^. Coutinho *et al*.^24^ evaluated the effects of a 3-h daily MS on visceral sensitivity, and demonstrated that neonatal stress predisposes adult rats to develop visceral hyperalgesia.

Nesfatin-1 is extensively located in the rat central nervous system; however, this mature peptide is not detectable in protein extracts from rat brain, and the nesfatin-1 receptor remains unknown^25^_._ Central or peripheral injection of nesfatin-1 in rats exerted an anorexic effect in a dose-dependent manner^26^. Furthermore, nesfatin-1 plays a regulatory role in the stress response^27^. Nesfatin-1 has been located widely in brain regions involved in stress mediation, such as the locus coeruleus, nucleus tractus solitarius (NTS), paraventricular nucleus (PVN), and nuclei of the amygdala and the DRN^12,16^. Moreover, stress has a marked impact on visceral sensitivity^28^. We previously demonstrated central nesfatin-1 contributes to the formation of visceral hypersensitivity through interactions with glucocorticoid and mineralocorticoid receptors in the amygdala in rat MS models^15^. As yet, it remains unknown whether DRN nesfatin-1 is critical for visceral sensation. Furthermore, the DRN is strongly involved in pain modulation and nociresponsive neurons are present within this structure^29,30^. Here, we show that MS rats exhibit elevated numbers of nesfatin-1-positive cells in the DRN. In addition, intra-DRN administration of anti-nesfatin-1/NUCB2 alleviated visceral hyperalgesia in MS model rats relative to vehicle-treated controls. In contrast, intra-DRN administration of nesfatin-1 produced visceral hyperalgesia with elevated AWR scores and EMG magnitude in normal adult rats. These results showed that nesfatin-1 in the DRN may be involved in the pathogenesis of MS-induced visceral hyperalgesia in rats.

5-HT is known to play a dominant role in the development of IBS through interaction with different receptors in the central or enteric nervous systems^31^. The DRN, a region rich in serotonergic neurons, represents cell bodies of the origin of the serotonergic system and projects widespread terminations into the forebrain and the brainstem^32^. MS induces an increase in 5-HT and cFos immunostaining in the DRN^10^, and there is higher turnover of 5-HT in the brainstem of MS rats as compared to NH animals^33^. In the present study, MS rats exhibit significantly elevated numbers of TPH-immunopositive cells in the DRN. The upregulated TPH expression in the DRN indicated that the biosynthesis of 5-HT itself may be increased, which in turn results in a dramatic elevation in the number of 5-HT neurons. These results indicate that MS results in hyperactivation of DRN 5-HT neurons.

Nesfatin-1 is colocalized with hypothalamic CRH neurons, brainstem noradrenaline, and 5-HT neurons^16^. Furthermore, intracerebroventricular administration of nesfatin-1 could activate CRH, noradrenaline, and 5-HT neurons^13^. We previously demonstrated that nesfatin-1 modulates visceral sensation in an IBS animal model, which may be mediated by the brain CRH/CRH1 signaling system^14^. As yet, it remains unknown whether nesfatin-1 leads to visceral hypersensitivity via 5-HT neurons in the DRN. In the present study, intra-DRN administration of anti-nesfatin-1/NUCB2 alleviated visceral hypersensitivity in MS model rats, accompanied by decreased 5-HT and TPH immunoreactivity in the DRN as compared with vehicle controls. These results demonstrated that the anti-hyperalgesic effect of anti-nesfatin-1/NUCB2 may be mediated by downregulated serotonergic activities in the DRN. However, intra-DRN administration of nesfatin-1 induced visceral hypersensitivity in normal rats, which correlated with elevated 5-HT and TPH immunoreactivity in the DRN. Taken together, nesfatin-1 could serve as a stress mediator—that is, stress may stimulate DRN nesfatin-1, which further activates DRN serotonergic neurons, evoking visceral hypersensitivity.

Hypersensitivity induced by visceral distension has been widely demonstrated in IBS, which can be associated with abnormal processing, modulation, and sensation of gut signals in the brain^34,35^. Drossman *et al*.^36^ discovered that poor clinical status in patients with IBS are associated with abnormal activation of the cingulate cortex. A meta-analysis of brain activation revealed that patients with IBS have exaggerated activation in the cerebral emotional arousal regions (anterior cingulate cortex and amygdala), and endogenous pain regulation regions (the midbrain) following visceral stimulation, as compared with controls^37^. These brain areas are heavily innervated by 5-HT neurons arising from the DRN^7^. The activation of the DRN 5-HT neurons in this IBS rat model may indicate their role in pain regulation and, moreover, because of their widespread projections, roles in other regions of the central nervous system.

Our study demonstrated that nesfatin-1 in the DRN has critical effect on visceral hyper -sensitivity in male maternally separated rats. MS induces colonic barrier dysfunction and mucosal inflammation in the colon^38^. However, nesfatin-1 and nesfatinergic neurons have anti-inflammatory actions^39^, peripheral colonic inflammation may stimulate central nesfatinergic system to ameliorate the inflammatory process. In the present study, we speculated that the neuroprotective effect of nesfatin-1 was not able to neutralize its role on visceral hypersensitivity.

In summary, Nesfatin-1 has a critical effect on visceral hypersensitivity, the underlying mechanism of which might be related to the activation of DRN 5-HT neurons.
